# Construction of Indium and Cerium Codoped Ordered Mesoporous TiO_2_ Aerogel Composite Material and Its High Photocatalytic Activity

**DOI:** 10.1002/gch2.201700118

**Published:** 2018-05-29

**Authors:** Ren‐Rong Zheng, Tian‐Tian Li, Hui Yu

**Affiliations:** ^1^ School of Chemistry and Environmental Engineering Changchun University of Science and Technology Changchun 130022 P. R. China

**Keywords:** composites, ordered mesoporous TiO_2_ aerogels, photocatalytic, Rhodamine B

## Abstract

In this study, ordered mesoporous In_2_O_3_‐CeO_2_/TiO_2_ aerogel composite material is fabricated via a sol–gel method. According to the preparation process of the aerogel, different weight percentages Ce(NO_3_)_3_ and In(NO_3_)_3_ are dissolved in the solvent, which would be completely dispersed in the porous gel when the system completely becomes gel. The prepared materials are used to degrade the Rhodamine B (Rh B) under visible light irradiation. 0.2 wt% In_2_O_3_‐0.2 wt% CeO_2_/TiO_2_ (In_0.2_‐Ce_0.2_/TiO_2_) sample has the highest degradation rate which reaches to 96.20%. When degradation time is continuously increased to 110 min, the degradation efficiency of In_0.2_‐Ce_0.2_/TiO_2_ sample is basically retained. The prepared In_0.2_‐Ce_0.2_/TiO_2_ sample has much better stability and reproducibility under visible light irradiation, the photocatalytic degradation efficiency of In_0.2_‐Ce_0.2_/TiO_2_ sample is still stable at more than 90% after the five times cycle.

## 1 Introduction

In recent years, the researches on various photocatalysts have attracted more and more attentions which has become a potential technical means in environmental and energy sustainable development, such as degradation of organic pollutants in waste water, organic synthesis, photolysis of water, photocatalytic treatment of desalination, and degradation of pharmaceuticals.^1^, ^2^, ^3^, ^4^, ^5^, ^6^ With the improvement of living standards, human beings pay more and more attention on their health and living environment. Water is the source of life and an important material basis for achieving sustainable development. With the industrial accelerated development, water pollution becomes more and more serious, water shortage has become an important factor to restrict economic development, and water pollution control becomes necessary. At present, the widest applied semiconductor photocatalysts mainly include titanium dioxide, zinc oxide, tin oxide and zinc sulfide.

TiO_2_ active composite photocatalysts are often used to degrade organic pollutants in air and wastewater. Under the irradiation of ultraviolet light, the mesoscopic structure anatase TiO_2_ could promote charge separation and improve the photocatalytic activity of materials.^7^, ^8^ Due to broad band width, TiO_2_ photocatalytic activity is limited in ultraviolet light range. At present, the researches on TiO_2_ photocatalyst are mainly focused on the increase of its photocatalytic activity in sunlight. Metal or nonmetallic ions doping is a much more effective method. Naldoni et al.^9^ introduced Ti^3+^ into TiO_2_, increased the oxygen vacancy concentration, decreased bandwidth of TiO_2_, and improved photocatalytic activity of TiO_2_ in visible light range. The Au nanoparticles were deposited on mesostructure anatase TiO_2_ by injection method, which could significantly improve the photocatalytic activity of material in visible light range.^10^ TiO_2_ is a promising photocatalytic degradation catalyst,^11^ but the pure TiO_2_ nanopowders have a small specific surface area, poor adsorption and light absorption capacity, and poor separability and reusability.^12^, ^13^ CO_2_ supercritical assisted liquid crystal soft template method was a new method, which could improve the separation and reusability of catalyst in a certain extent. However, the active site (TiO_2_) was less, its catalytic activity was relatively low.^14^ The matrix materials play a very important role in catalyst design. The matrix materials should have the higher adsorption performance, the greater TiO_2_ loading quantity, and the more active sites. The specific surface area can be enhanced by synthesizing nanostructured TiO_2_,^15^, ^16^, ^17^ or load TiO_2_ on a high specific surface area substrate.^18^, ^19^, ^20^ TiO_2_ was doped on fibrous SiO_2_ substrate, which has higher photocatalytic activity than in Santa Barbara Amorphous (SBA)‐15 or mesoporous crystalline material (MCM)‐41 mesoporous materials.^21^, ^22^, ^23^

CeO_2_ is one of the most active rare earth metal oxides with a band gap of 2.92 eV. It has a high optical transparency in visible region, and possesses a high capacity to store oxygen.^24^, ^25^ CeO_2_ can be used for photocatalytic degradation of organic pollutants in wastewater.^26^, ^27^ The metal ions were doped into TiO_2_, which could improve the electron–hole separation efficiency,^28^ and improved photocatalytic efficiency.^29^, ^30^, ^31^, ^32^, ^33^, ^34^ The introduction of CeO_2_ into TiO_2_ framework can effectively extend the visible light response of TiO_2_. Because of its low specific surface area, CeO_2_/TiO_2_ catalyst usually has the lower photocatalytic activity. Many researchers have focused on preparation of mesostructured CeO_2_/TiO_2_, which have a large surface area and controllable pore size to improve its photocatalytic activity.

TiO_2_‐In_2_O_3_ composite photocatalysts have been explored by many researchers. In_2_O_3_, a semiconductor with a direct band gap of 3.6 eV and an indirect band gap of 2.8 eV, is an efficient sensitizer to extend the absorption spectra of oxide semiconductor photocatalysts from the UV region to the visible region.^35^ The electrochemical experiment showed that the molecular O_2_ was reduced on the In_2_O_3_ surface rather than on the TiO_2_ surface at a markedly lower overvoltage.^36^ The amount of hydroxyl groups on TiO_2_ surface were greatly increased after doping with indium. The super hydrophilic In^3+^ doped TiO_2_ was also reported by Eshaghi et al.^37^ Gonzaílez et al.^38^ reported the synthesis, characterization and photocatalytic properties of In_2_O_3_‐TiO_2_ catalysts. The large number of structural defects enhanced the acidity and adsorption of pollutants, and H_3_O^+^ produced much more number of •OH radicals.

In this study, we prepared indium and cerium oxides codoped ordered mesoporous CeO_2_‐In_2_O_3_/TiO_2_ aerogel composite material by sol–gel without templates method. TiO_2_ light absorption band edge distributed in ultraviolet region, the doping of indium and cerium dopants shifted the light absorption band edge to the visible light region, and enhanced the photocatalytic activity by efficiently separating charge carriers (electrons/holes). This study was quite different from the past reported articles in preparation and photocatalytic. The aerogel technology was used to prepare ordered mesoporous CeO_2_‐In_2_O_3_/TiO_2_ aerogel material, which has never been report on indium and cerium oxides codoped ordered mesoporous TiO_2_. The structure of the prepared material was characterized by X‐ray diffraction (XRD), scanning election microscope (SEM), transmission electron microscopy (TEM), N_2_ adsorption–desorption method, energy dispersive X‐ray spectrometer (EDS), and UV–vis spectrophotometer. The spectroscopic characterization of the CeO_2_‐In_2_O_3_/TiO_2_ photocatalysts and their photocatalytic activities in degradation of Rhodamine B were also investigated under visible light irradiation.

## 2 Results and Discussion

### 2.1 Structural Characterizations

**Figure 1:**
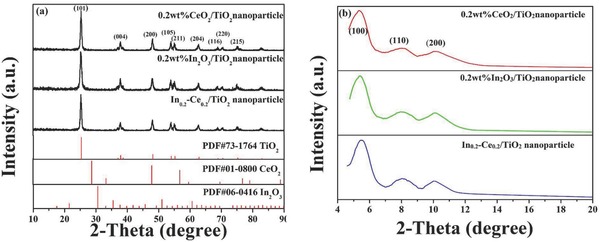
a) Wide‐angle XRD and b) small‐angle XRD patterns of 0.2 wt% CeO_2_/TiO_2_, 0.2 wt% In_2_O_3_/TiO_2_ and In_0.2_‐Ce_0.2_/TiO_2_ samples.

**Figure** **1** shows wide‐angle and small‐angle XRD (SAXS) patterns of the prepared samples. From Figure 1a, the peaks at 25.3°, 37.8°, 47.9°, 53.8°, 55.1°, 62.7°, 68.7°, 70.3°, and 75.0° could be attributed to (101), (004), (200), (105), (211), (204), (116), (220), and (215) crystal plane diffraction of anatase phase TiO_2_ (The powder diffraction file (PDF) No. 73‐1764). Moreover, the (101) diffraction peak of the anatase phase TiO_2_ was only observed in the samples, and (100) diffraction peaks of the rutile phase TiO_2_ was not showed. It was concluded that the TiO_2_ was pure anatase phase structure in prepared materials. Because of low dopant concentration, the characteristic diffraction peaks of CeO_2_ and In_2_O_3_ species were not observed. It was difficult for the eight coordinated Ce in cubic CeO_2_ lattice to replace six coordinated Ti in tetragonal TiO_2_ lattice.^39^ Hence, Ce ions might be highly dispersed in porous of ordered mesoporous TiO_2_ in the form of metal oxides. From Figure 1b, all prepared samples exhibited three peaks near 2θ = 5.4°, 2θ = 8.1°, and 2θ = 10.0°, which could be attributed to (100), (110), and (200) crystal face diffraction peaks of the ordered mesoporous. The diffraction peaks of In_0.2_‐Ce_0.2_/TiO_2_ sample slightly shifted to a higher angle, which revealed small shrinkage of skeleton structure.^40^

**Figure 2:**
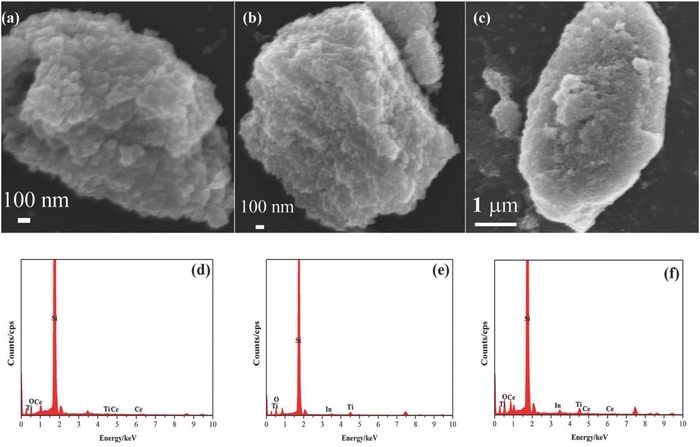
SEM and EDS images of the prepared samples: a,d) 0.2 wt% CeO_2_/TiO_2_, b,e) 0.2 wt% In_2_O_3_/TiO_2_, and c,f) In_0.2_‐Ce_0.2_/TiO_2_ samples.

SEM images of prepared 0.2 wt% CeO_2_/TiO_2_, 0.2 wt% In_2_O_3_/TiO_2_ and In_0.2_‐Ce_0.2_/TiO_2_ samples were shown in **Figure** **2**a–c. The prepared composites showed a gel block structure, which were constituted with heterogeneous spherical gel particles. The single irregular spherical particle sizes were about 100, 82, and 50 nm, respectively. EDS images of the prepared samples are shown in Figure 2d–f. The signal of Si was introduced from silicon slice, which was used in test procedure. Ti, O, In, and Ce peaks were obviously found in the energy spectrum, which confirmed that In and Ce existed in TiO_2_ matrix. From EDS analysis results, the atomic percentages of Ti, O, In, and Ce were shown in **Table** **1**.

**Table 1:** The atomic percentages of Ti, O, In, and Ce

Catalysis	Atomic percentage of Ti	Atomic percentage of O	Atomic percentage of In	Atomic percentage of Ce
0.2 wt% CeO_2_/TiO_2_	62.31	37.28	0	0.41
0.2 wt% In_2_O_3_/TiO_2_	63.17	36.39	0.44	0
In_0.2_‐Ce_0.2_/TiO_2_	60.22	38.91	0.42	0.45

**Figure 3:**
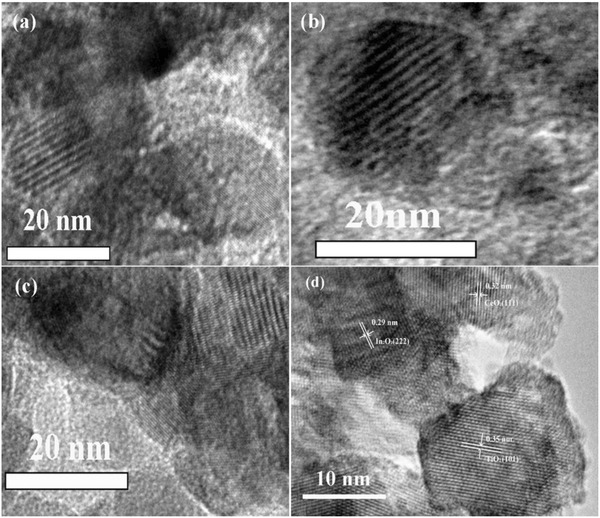
TEM images of the prepared a) 0.2 wt% CeO_2_/TiO_2_, b) 0.2 wt% In_2_O_3_/TiO_2_, c) In_0.2_‐Ce_0.2_/TiO_2_ samples, and d) HRTEM image of In_0.2_‐Ce_0.2_/TiO_2_ sample.

**Figure** **3**a–c shows TEM images of the prepared samples, which were taken with beam direction perpendicular to the pores. It could be seen that a large number of ordered porous structures were distributed on the surface of prepared samples. Figure 3d shows HRTEM image of the prepared In_0.2_‐Ce_0.2_/TiO_2_ sample. The lattice fringes with lattice space of 0.35 nm were clearly seen, which were corresponding to (101) lattice plane of TiO_2_ (Joint Committee on Powder Diffraction Standards (JCPDS) PDF No. 73‐1764). The lattice fringes with lattice space of 0.32 nm were (111) lattice plane of CeO_2_ (JCPDS PDF No. 01‐0800), and lattice fringes with lattice space of 0.29 nm were (222) lattice plane of In_2_O_3_ (JCPDS PDF No. 06‐0416).

**Figure 4:**
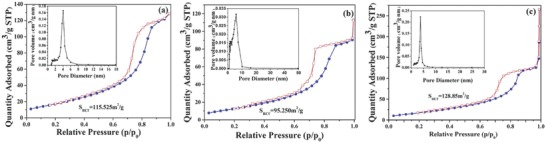
N_2_ adsorption–desorption isotherms and pore size distribution curve of a). 0.2 wt% CeO_2_/TiO_2_, b) 0.2 wt% In_2_O_3_/TiO_2_, and c) In_0.2_‐Ce_0.2_/TiO_2_ samples.

**Figure** **4** shows N_2_ adsorption–desorption isotherms and pore size distribution curve of the prepared samples. The isotherms were type‐IV isotherm with a H1 hysteresis loop, which were the typical characteristic of mesoporous materials with cylindrical pores. The adsorption branch showed an uptake at relative pressure 0.4–0.8 range, it could be attributed to capillary condensation of nitrogen into mesoporous, which was also a characteristic feature of mesoporous materials.^41^ From Figure 4 illustration, the samples had a narrow porous size distribution. In this study, BET (Brunner–Emmett–Teller) method and BJH (Barrett–Joyner–Halenda) method were used to calculate the specific pore parameters. All data were derived from desorption branch of the nitrogen adsorption–desorption isotherm. The average pore size, specific surface area and pore volume were shown in **Table** **2**. With increase of dopant concentration, the particle size decreased, and the surface area increased, which was contributed to refined crystal grain effect of rare earth. At higher dopant concentration, the highly dispersed In_2_O_3_ and CeO_2_ obstructed the pores of TiO_2_, which resulted in the decrease of porous size.^36^

**Table 2:** Crystallite size and porous parameter of the prepared samples

Catalysis	Crystallite size [nm]	Specific surface area [m^2^ g^−1^]	Pore volume [cm^3^ g^−1^]	Pore diameter [nm]
Meso‐TiO_2_	18.7	51.517	0.1471	3.46
0.2 wt% CeO_2_/TiO_2_	16.4	115.525	0.3642	4.17
0.2 wt% In_2_O_3_/TiO_2_	13.8	95.250	0.1863	5.92
In_0.1_‐Ce_0.1_/TiO_2_	11.4	97.735	0.4734	4.24
In_0.2_‐Ce_0.2_/TiO_2_	9.2	128.85	0.4298	3.80
In_0.3_‐Ce_0.3_/TiO_2_	8.9	125.74	0.4178	3.68

**Figure 5:**
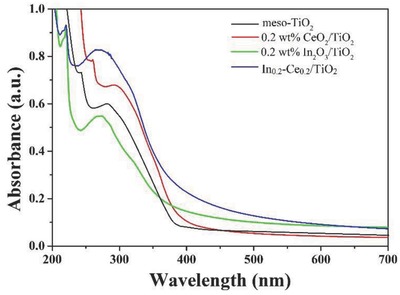
UV–vis absorption spectra of the prepared samples.

UV–vis absorption spectra of the prepared samples were showed in **Figure** **5**. The prepared TiO_2_ aerogel had much better absorption ability to ultraviolet light, which could not absorb the visible light. The absorption band appeared at 350–450 nm, which had an absorption in the visible range. For a semiconductor, the band edge can be calculated via the follow formulas

(1) $$ah\nu \;\;=\;\;A\;{\left(h\nu \;\;-\;\;E_{g}\right)}^{n/2}$$

(2) $$E_{g}=1240/\lambda_{g}$$

where *α, ν, E*_g_, and *A* are absorption coefficient, light frequency, band gap, and a constant, respectively. It should be noted that the *n* is determined by the type of optical transition of a semiconductor in Equation (1), which should be 1 for the TiO_2_ due to its indirect semiconductor nature. Equation (2) is applied to estimate the *E*_g_ of the TiO_2_, in which λ_g_ refers to the wavelength of the absorption edge. The onset of absorption for TiO_2_, 0.2 wt% CeO_2_/TiO_2_, 0.2 wt% In_2_O_3_/TiO_2_ and In_0.2_‐Ce_0.2_/TiO_2_ samples were observed at 392, 396, 412, and 436 nm, respectively. The band gap energy values were calculated using Kubelka–Munk equation, which were 3.16, 3.13, 3.01, and 2.84 eV for TiO_2_, 0.2 wt% CeO_2_/TiO_2_, 0.2 wt% In_2_O_3_/TiO_2_ and In_0.2_‐Ce_0.2_/TiO_2_ samples, respectively. It revealed that the codoping of indium and cerium showed much better red shift than single‐doped indium and cerium samples.

**Figure 6:**
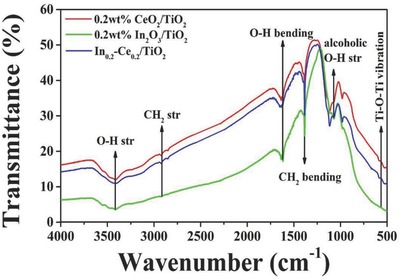
FT‐IR spectra of 0.2 wt% CeO_2_/TiO_2_, 0.2 wt% In_2_O_3_/TiO_2_, and In_0.2_‐Ce_0.2_/TiO_2_ samples.

The Fourier transform infrared spectroscopy (FT‐IR) spectra of meso‐0.2 wt% CeO_2_/TiO_2_, 0.2 wt% In_2_O_3_/TiO_2_ and In_0.2_‐Ce_0.2_/TiO_2_ samples were shown in **Figure** **6**. In the spectrum of prepared material, the broad band around 3395 cm^−1^ was due to O—H stretching vibration of surface adsorbed water. The bending vibration of water appeared at 1624 cm^−1^. The —CH_2_ stretching vibration of template showed a small band at 2926 cm^−1^. The intense sharp band at 1375 cm^−1^ was attributed to —CH_2_ bending vibration of the n‐hexane solution. The band at about 1100 cm^−1^ was due to C—O stretching vibration of alcohol used in the synthesis. The broad band at 617 cm^−1^ was ascribed to the strong stretching vibration of Ti—O—Ti bonds.^42^

### 2.2 Photocatalytic Performance

**Figure 7:**
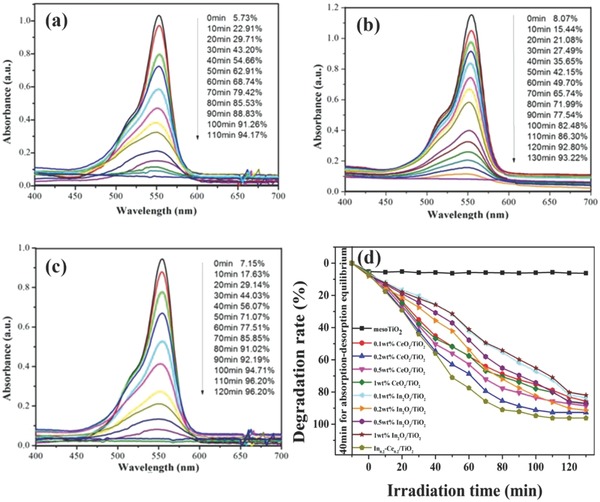
Degradation curves to Rh B of the prepared a) 0.2 wt% CeO_2_/TiO_2_, b) 0.2 wt% In_2_O_3_/TiO_2_, and c) In_0.2_‐Ce_0.2_/TiO_2_ samples and d) degradation rate‐time curves to Rh B of the prepared samples with different ratio CeO_2_ and In_2_O_3_ under visible light.

**Figure** **7** shows photocatalytic degradation curves and degradation rate‐time curves of the prepared samples. When the degradation time was 110 min, the degradation rates of 0.2 wt% CeO_2_/TiO_2_, 0.2 wt% In_2_O_3_/TiO_2_ and In_0.2_‐Ce_0.2_/TiO_2_ samples respectively reached 94.14%, 86.30% and 96.20%, and the degradation efficiency basically remained unchanged with the degradation time continues to increase. It could be seen that the doped amount of CeO_2_ or In_2_O_3_ had a great effect on the degradation of Rh B. There was almost no degradation for TiO_2_ aerogel under the visible light. It is because that TiO_2_ has only the absorption ability to ultraviolet light. From Figure 7d, it showed that the best doping concentration of Ce or In was both 0.2 wt%, and the degradation efficiency of In_0.2_‐Ce_0.2_/TiO_2_ sample was better than In*_z_*‐Ce_0.2_/TiO_2_ (*z* = 0, 0.1, 0.5, 1).

**Figure 8:**
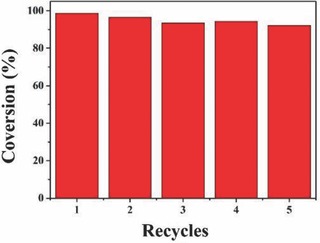
The cycling stability test result of In_0.2_‐Ce_0.2_/TiO_2_ catalyst.

**Figure** **8** shows Rh B degradation cycling stability test of In_0.2_‐Ce_0.2_/TiO_2_ sample under visible light. During recycling experiment, the catalyst was collected by centrifugation, washed with deionized water, dried at 60 °C, and reused in the next cycles. The photocatalytic degradation efficiency was still stable at more than 90% after five times cycle. It indicated that the prepared sample had better stability and reproducibility under visible light irradiation.

### 2.3 Photocatalytic Mechanism

It is well known that indium and cerium doped titanium dioxide plays an important role in the visible light absorption.^43^, ^44^, ^45^, ^46^ The prepared samples can be activated by visible light, and result in the more electrons and holes in photocatalytic oxidation–reduction reactions. The doping of indium ions results in the formation of the surface energy level at the electron energy level below the conduction band (CB) of TiO_2_ at 0.3 eV. Wang et al.^44^ reported the electron transition from the titania valence band (VB) to surface state level. The photocatalyst in visible region is activated, which can be attributed to the reduced bandgap. Photogenerated electrons tend to accumulate at the surface state level, and photogenerated holes accumulate on the TiO_2_ surface. The large mesoporous make the reactant molecules to easily access the active sites inside the pores, and provide the more pathways for the reactants to enter and the products to escape. Due to the charge imbalance on the TiO_2_ solid surface, the adsorption is also increased. When Ti^4+^ cations are substituted by In^3+^ cations in the titania lattice surface, a charge imbalance is produced by —Ti—O—In—O—Ti— framework. The negative charges at the solid surfaces improved the adsorption of H_3_O^+^ as well as cationic Rh B species.^38^, ^45^ The formation of surface energy level is a main reason to enhance the photocatalytic activity of visible light. With indium doping, the excess negative charges on the —Ti—O—In—O—Ti— surface induce the adsorption of H_3_O^+^, and introduces the Brønsted acidity. Thus, the surface ‐OH groups has also been enhanced. During the photocatalytic reaction, Rh B molecules adsorb on the surface active site of In_0.2_‐Ce_0.2_/TiO_2_, which are immediately oxidized by •OH radicals. At the same time, the photogenerated electrons accumulate at the surface state energy level which are captured directly by Ce^4+^ ions, and transfer to the adsorbed O_2_ molecules on the surface to form O_2_•^−^ active species.^46^, ^47^

The conduction band of TiO_2_ or the surface state energy level is more negative than the reduction potential of Ce^4+^ to Ce^3+^ (+1.61 eV). The excitation energy of the electrons at CB or surface level can reduce Ce^4+^ to Ce^3+^ under visible light irradiation. The trapped electrons on Ce^4+^ site can be easily transferred to adsorbed oxides (O_2_•^−^) on the surface of In_0.2_‐Ce_0.2_/TiO_2_ catalyst. Under the protonation, the superoxide anion radicals (O_2_•^−^) generates the hydroperoxyl radicals (HO_2_•) which subsequently produces hydroxyl radicals (•OH). In terms of cerium doping, the relative mobility of oxygen on the surface of TiO_2_ is much higher.^25^, ^45^ The VB of TiO_2_ also has a potential of +2.9 eV, which is more positive than the oxidation potential of Ce^3+^ to Ce^4+^ (−1.61 eV). These cerium oxide ion species can carry the species of hydroxyl groups, and produce a large number of highly active hydroxyl radicals (•OH).^43^ Ce^3+^ and Ce^4+^ coexist enhance the photocatalytic activity by inhibiting electron–hole recombination. The excitation of electrons and holes can be carried out by Ce^4+^ and Ce^3+^ ions through the following process^48^

(3) $${\mathrm{Ce}}^{4+}+e^{-}\to {\mathrm{Ce}}^{3+}$$

(4) $${\mathrm{Ce}}^{3+}+O_{2}\to {\mathrm{Ce}}^{4+}+O_{2}{•}^{-}$$

(5) $${\mathrm{Ce}}^{3+}+h^{+}\to {\mathrm{Ce}}^{4+}$$

(6) $${\mathrm{Ce}}^{4+}+{\mathrm{OH}}^{-}\to {\mathrm{Ce}}^{3+}+•\mathrm{OH}$$

(7) $$\mathrm{Rh}\;B+•\mathrm{OH}\to {\mathrm{CO}}_{2}↑+\;H_{2}O$$

In addition, the potential of VB in TiO_2_ (+2.9 V) is more positive than the reduction potential of OH^−^ to •OH (+1.9 eV). The VB of TiO_2_ has the tendency to accept more electrons, and the photogenerated holes are easily oxidized by the adsorbent OH^−^ to form •OH radicals, which are a strong oxidizing agent to decompose of Rh B. The electron–hole recombination is suppressed, and the quantum yield of photocatalysis is enhanced by indium and cerium doping. Visible light irradiation produces a large amount of •OH radical, and improves the photocatalytic activity.

**Figure 9:**
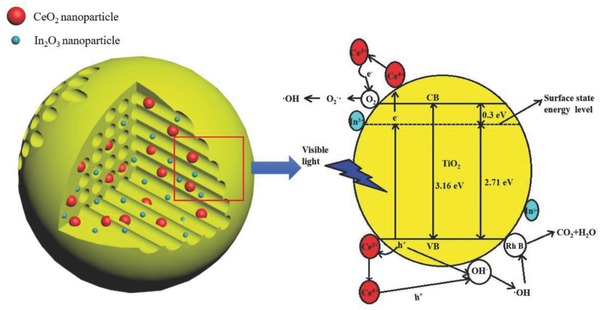
Photocatalytic degradation mechanism diagram of the In_0.2_‐Ce_0.2_/TiO_2_.

The proposed photocatalysis mechanism of In_0.2_‐Ce_0.2_/TiO_2_ is shown in **Figure** **9**. In^3+^ and Ce^4+^/Ce^3+^ play an important role in improving the photocatalytic activity of TiO_2_ matrix. The surface energy level is below conduction band of TiO_2_ (0.3 eV), it dues to the O–In–Ce species on the surface of the titanium dioxide, and the visible light stimulates the valence band electrons to the surface state level. The excited electrons are transferred by holes of Ce^4+^ and Ce^3+^ to the hydroxyl groups of the adsorbed oxygen molecules to produce a large amount of superoxide anion radicals and highly active hydroxyl radicals. The charge transfer efficiency and visible activity of the photocatalyst are enhanced by the doped Ce^4+^/Ce^3+^. In addition, the adsorbed H_3_O^+^ and cationic Rh B species on TiO_2_ surface are improved by In^3+^, which due to the negative charge on the solid surface. The large number of O_2_•^−^ and •OH efficiently degrade the adsorbed Rh B molecules, and ultimately enhance the photocatalytic activity.

## 3 Conclusions

In this study, the ordered mesoporous In_2_O_3_‐CeO_2_/TiO_2_ aerogel composite material was fabricated via sol–gel method. The prepared In_2_O_3_‐CeO_2_/TiO_2_ composite had anatase TiO_2_ crystal structure. The monodisperse particle size was about 8–21 nm, and the prepared composite material retained the porous structure of the host material. The prepared materials were used to the degradation of the Rh B. Among all the synthesized photocatalysts with different dopant concentration, In_0.2_‐Ce_0.2_/TiO_2_ nanocomposite showed the highest photocatalytic activity, which could be attributed to the increased surface area with sufficiently porous structure. The large number mesoporous provided the more active sites inside pores. The degradation efficiency of In_0.2_‐Ce_0.2_/TiO_2_ sample reached 96.20% in 110 min, which had the better stability and reproducibility under visible light irradiation, the photocatalytic degradation efficiency was still stable at more than 90% after the five times cycle.

## 4 Experimental Section

*Materials*: The tetrabutyl titanate (A.R., Shanghai Chemical Pharmaceutical Co., Ltd. China), cerium (III) nitrate hexahydrate (A.R., Tianjin Guangfu Fine Chemical Research Institute), and indium (III) nitrate hydrate (A.R., Sinopharm Chemical Reagent Co., Ltd) were used as the precursors of Ti, Ce, and In, respectively. Aceticacid (A.R., Shanghai Chemical Pharmaceutical Co., Ltd. China) was used as catalyzer. All reactants were analytical grade and used without further purification. Deionized water and absolute alcohol were used throughout. Rhodamine B (A.R., Tianjin Guangfu Fine Chemical Research Institute) was used as the model pollutant for the degradation study.

*Synthesis of Ordered Mesoporous CeO_2_‐In_2_O_3_/TiO_2_ Aerogel Catalysts*: According to the prepared process of aerogel, the different weight percentages Ce(NO_3_)_3_ and In(NO_3_)_3_ were dispersed in the solvent. The solvent would be volatilized, and Ce_2_O_3_ and In_2_O_3_ would be almost completely dispersed in the porous of the obtained porous TiO_2_ substrate when the material was heat treated. The specific synthesis process as follows: 8.7 mL tetrabutyl titanate and 6.2 mL ethanol were mixed with a certain percentage of Ce(NO_3_)_3_ ⋅ 6H_2_O and In(NO_3_)_3_ ⋅ 4.5H_2_O under stirring, the solution was signed as A solution. 3.1 mL acetic acid solution was mixed with 3.8 mL of distilled water with stirring, the solution was signed as B solution. The solution B was rapidly added into the solution A, and formed the gel. The gel was aged at room temperature for 24 h, and then continually aged in water at 70 °C for 2–4 d. The aged gel was soaked in *n*‐hexane solution at 50 °C, and *n*‐hexane solution was replaced three times in 48 h. The gel was dried at 100 °C for 24 h, and obtained the ordered mesoporous CeO_2_‐In_2_O_3_/TiO_2_ aerogel catalysts.

*Photocatalytic Experiments*: Photocatalytic activity of the prepared samples for degradation of Rh B was measured under visible light irradiation. The xenon lamp (500 W) was used as the visible light source, which distanced the solution surface about 5 cm. In course of experiment, 50 mg prepared samples were thrown into mixed solution of 5 mL Rh B solution (0.1 g L^−1^) and 95 mL distilled water, and the mixture was stirred in the dark for 1 h, and the photocatalyst with Rh B solution reached an adsorption–desorption equilibrium. Then, the solution was placed in visible light to irradiate, 4 mL of the mixed solution was removed every 5 min, and centrifuged to remove the photocatalyst whose concentration was determined. All of measurements were carried out at room temperature.

*Characterization Method*: X‐ray powder diffraction (X‐ray diffraction (XRD)) patterns were collected on a Siemens D5005 diffractometer using Cu‐*K*_α_ radiation (λ = 1.5418 Å and operating at 30 kV and 20 mA). TEM images were taken on a JEOL 2010 TEM instrument. The SEM images were taken on a JEOL JSM‐5600L instrument, the compositions of samples were examined by Oxford ISIS‐300 EDS attached to the scanning electron microscope (SEM). The UV–vis absorption spectra for the evaluation of photocatalytic properties were measured through the Shimadzu UV‐3600 spectrophotometer. The UV–vis spectrophotometer was used to measure the degradation degree of Rh B through N4 spectrophotometer. All the measurements were tested at room temperature. Bet surface area (BET) and pores parameters of In_2_O_3_‐CeO_2_/TiO_2_ aerogel composites were identified by physical adsorption of nitrogen on a Micromeritics ASAP2010M volumetric adsorption analyzer. The In_2_O_3_‐CeO_2_/TiO_2_ aerogel composites were degassed in vacuum at 573 K for 3 h before measurement. Pore size was computed using the BJH method. The FT‐IR spectrum was recorded on Perkin Elmer FT‐IR spectrometer using KBr pellet technique. The pellet was scanned at 4 cm^−1^ resolution in the range of 4000–400 cm^−1^.

## Conflict of Interest

The authors declare no conflict of interest.
